# Poor-Law Expenditure

**Published:** 1905-09-09

**Authors:** 


### POOR-LAW EXPENDITURE.

Miss Edith Sellers' article in the *Nineteenth Century*, on "How Poor-law Guardians Spend their Money" is sure to attract attention at a time when most people are groaning at the poor-rate and anticipating an increase in it during the winter. Unfortunately Miss Sellers' article is founded exclusively on the study of one rather small poor-law area, and considering the great liberty allowed the guardians of every district, we cannot accept this as absolutely typical of the whole. Perhaps greater uniformity is desirable, but as things stand, it is not possible to draw safe inferences as to the management of the poor as a whole from a study limited, like Miss Sellers', to a comparatively small district. Had Miss Sellers given the statistics of a number of unions, both urban and rural, in different parts of the country, we should have regarded such a summary as helpful. We cannot say the same of an estimate of one small, unnamed parish.

This does not mean that we do not agree in some things with Miss Sellers. She had great difficulty in getting at her facts owing to the reports published by the union with which she deals being incomplete in many points. It is unfortunate that there is no uniform style of report adopted by all unions, and the Local Government Board would do a service both to Poor-law officials and to ratepayers if they would draw up something akin to the "Uniform System of Accounts" now adopted by so many hospitals, and bid the clerks of all poor-law unions keep their accounts in one fashion. At present the best attempts to compare the expenditure of one district with another are apt to be baffled by the absence in the published reports of any adequate basis of comparison. Thus —to go no further than London—if we want to know what food is allowed an able-bodied pauper, we can get the requisite information from the reports of Wandsworth, Fulham, and Lewisham, but not from that of Paddington, which gives only the dietary tables for infirmary patients. On the other hand, Paddington tells at a glance how the expenditure on the relief of the poor has developed during the last 50 years, for which information we look in vain in the reports of Wandsworth and Lewisham. Wandsworth tells those who read its report to what kinds of work it sent the children brought up at its schools. Lewisham shares these schools, but gives its ratepayers no such information as to the after history of the children whose bringing up they have paid for. There is hardly a single point which is not dealt with in one report and omitted in another. Thus any attempt to find out exactly to what purpose the rates are spent in different unions is exceedingly difficult, if not absolutely impossible. Until there is a uniform and complete report given by all unions for public perusal, efforts like those of Miss Sellers to say what is right and what is wrong in Poor-law expenditure will very largely prove futile. They may excite attention when the public is in the mood for it, but they will do little to help the ratepayers in different districts to control the employment of the money they are compelled to give.

Miss Sellers' chief complaint is the very comprehensible one that so much more is spent in administration than on the relief of the poor. This is exactly what is most apt to irritate the ratepayer, and with good reason. In Miss Sellers' union some of the items of expenditure seem incomprehensible—for example, the expenditure in three years of over £3,000 on repairing a laundry, and the payment of a retaining fee of £200 a year to a solicitor, who is paid in addition for the work he does. But she would be wrong in assuming that these things are universal. On the other hand, there are items which, though they appear large are disproportionate only because the ground they cover is so small. Speaking generally it is cheaper to deal with things on a large scale. Thus where there is a workhouse there must be a workhouse master and matron, whether the building accommodates one hundred inmates or five hundred. In the former case the salaries may be a little smaller; but, as board, lodging, and washing are included as part of the emoluments of the position, the difference in expense will be by no means proportionate to the difference in the numbers in the two institutions. The same number of paupers, scattered through half a dozen different workhouses, will cost more per head for administration expenses than they would if they were all gathered under one roof. To say this is no more than to say that if a hundred families, each of whom keep one servant, were to live together in a big hotel, they could have more efficient service with a smaller number of attendants. But it does not follow that it is always practicable or even desirable so to herd people in crowds. But it must always be remembered that in a small institution administration expenses are likely to bear a larger ratio to maintenance expenses than in a larger one, and the fact that Miss Sellers has taken only one small workhouse from which to draw her inferences makes them less valuable than they might have been had they been founded on more extensive investigations. Her ideas as to the combination of duties of the officials will surprise those who know what Poor-law work is—how trying, how thankless, how little appreciated. She thinks that the labour-master and labour-mistress of the workhouse might act as master and mistress of the casuals also. But the labour-master has to start work in the early morning—and those who know what inmate labour is will understand that the task of supervising it is no sinecure, and it is just when his day's work is done that the casuals begin to come in. Should he, having disposed of his resident paupers, be asked to supervise the washing of vagrants who drop in just as they like up to a late hour at night, and the placing of them in their respective cells. Then, while he is looking after his vagrants in the morning, and setting them to the tasks allotted them, who is to look after his other paupers? The thing might be done, but not without some sacrifice of that automatic regularity on which depends the getting through of the necessary work. Miss Sellers is very scornful regarding the laundry, which she thinks is less needed for the paupers' "bits of things" than for the officials' collars. But the paupers' "bits of things" include, besides their wearing apparel—which is changed as frequently as it is in most middle-class families—their bed-linen, towels, etc. For even the 174 indoor paupers with whom she deals this means a pretty large weekly wash, and for the laundry superintendent something more than a sinecure. In many particulars one could, while admitting the accuracy of Miss Sellers' figures, dispute the justice of her conclusions. But certainly one thing appears clearly—namely, that a pauper is an expensive luxury to the community that supports him. Wherefore, we welcome every attempt to keep him a self-supporting member of the community, and to restore him to that position when he has lost it. But before accepting the conclusion that Guardians either carelessly or wilfully waste the ratepayers' money, we should want a wider survey of the question than is put before us in the *Nineteenth Century*.

